# Antioxidant and Anti-Inflammatory Activities from Optimum Formula of *Spatholobus littoralis* Hassk. and *Sauropus androgynus* L.: In Vitro and In Silico Studies

**DOI:** 10.3390/cimb47120969

**Published:** 2025-11-21

**Authors:** Rut Novalia Rahmawati Sianipar, Dyah Iswantini, Charlena Charlena, Setyanto Tri Wahyudi, Joni Prasetyo, Trivadila Trivadila

**Affiliations:** 1 Department of Chemistry, Faculty of Mathematics and Natural Sciences, IPB University, Bogor 16680, West Java, Indonesia; rutnovaliasianipar@apps.ipb.ac.id (R.N.R.S.); dyahis@apps.ipb.ac.id (D.I.); charlena@apps.ipb.ac.id (C.C.); trivadila@apps.ipb.ac.id (T.T.); 2 Tropical Biopharmaca Research Center, IPB University, Bogor 16128, West Java, Indonesia; 3 Department of Physics, Faculty of Mathematics and Natural Sciences, IPB University, Bogor 16680, West Java, Indonesia; 4 Research Center for Chemistry, National Research and Innovation Agency (BRIN), Building 452, Serpong, South Tangerang 15314, Banten, Indonesia; joni002@brin.go.id

**Keywords:** anti-inflammatory, antioxidant, Simple Lattice Design, *Sauropus androgynus* L., *Spatholobus littoralis* Hassk.

## Abstract

This study aimed to optimize a formulation of *Spatholobus littoralis* Hassk. stems and *Sauropus androgynus* L. leaves using Simple Lattice Design (SLD). In this context, the response variable was DPPH (2,2-Diphenyl-1-picrylhydrazyl) antioxidant activity, while the optimum extract concentration of the two plants served as the experimental factor. Subsequently, the optimum formula was investigated for its in vitro anti-inflammatory activity against COX-2 (cyclooxygenase-2) and through in silico study. Molecular docking on the COX-2 receptor (PDB ID: 5IKQ) from the secondary metabolite profile was validated for the optimum formula. The formulation recommended by SLD comprised a 1:1 mixture of 70% ethanol extract of *S. littoralis* Hassk. stems and *S. androgynus* L. leaves. This optimum formula had an IC_50_ value of 108.70 µg/mL for the DPPH antioxidant with a synergistic effect due to the production of higher COX-2 inhibitory activity (73.05 ± 2.36%) than the single extract at 100 µg/mL. Daidzein (−8.514 kcal/mol), (10E,12Z)-9-Hydroperoxy-10,12-octadecadienoic acid (−7.604 kcal/mol), arteannuic acid (−7.114 kcal/mol), L-Proline,4-hydroxy-5-oxo-4-(tetrahydro-2,3,4-trihydroxy-2-furanyl)- (−6.480 kcal/mol), and Androst-2-en-17-amine,4,4-dimethyl-N-(2-phenylethyl)-, (5.alpha.)- (−5.440 kcal/mol) were the five compounds from the 70% ethanol extract of *S. littoralis* Hassk. stems and *S. androgynus* L. leaves that strongly bound 5IKQ. These compounds were obtained from five groups of compounds, namely flavonoids, fatty acids, terpenoids, amino acid derivatives, and amine derivatives. The formulation of *S. littoralis* Hassk. and *S. androgynus* L. extract has potential as an antioxidant and anti-inflammatory agent.

## 1. Introduction

Compounds or molecules with one or more unpaired electrons in the outer orbit are known as free radicals. These substances, which include hydroxyl (HO·), superoxide (O_2_·), alkoxy radicals (RO·), and peroxy radicals (ROO·), are referred to as reactive oxygen species (ROS). Free radicals, such as nitrogen dioxide (·NO_2_) and nitric oxide (·NO), are classified as reactive nitrogen species (RNS). Compounds become reactive with unpaired electrons. This leads to lipid peroxidation, protein denaturation, and DNA (deoxyribonucleic acid) fragmentation [1,2].

Free radicals can originate from both internal (endogenous) and external (exogenous) sources. Endogenous free radicals are produced within the human body through normal metabolism, which generates over 90% of oxygen during the food oxidation process to produce energy. In this context, mitochondria are the primary site for the production and elimination of ROS in cells. ROS, particularly H_2_O_2_, decrease the activity of the respiratory chain complex, causing oxidative phosphorylation and reducing adenosine triphosphate (ATP) energy supply to maintain normal functioning of cells. Damaged mitochondria significantly contribute to the harmful impact of ROS on osteogenesis. ROS reduces alkaline phosphatase (ALP) and collagen I expression while also inhibiting mineralization during bone marrow mesenchymal stem cells’ (BM-MSCs) and osteoblast precursors’ osteogenic development [3]. Exogenous sources of free radicals include cigarette smoke, environmental pollution, radiation, drugs, ozone depletion, chemicals, alcohol consumption, and pathological microorganisms. Oxidative stress increases the number of osteoclasts (bone resorption cells) while decreasing the number of osteoblasts (bone formation cells) [4,5].

Oxidative stress drives inflammation and is tightly regulated by the immune response to combat mechanical, chemical, and thermal injury. Nonsteroidal anti-inflammatory drugs block the enzyme cyclooxygenase (COX), which is responsible for converting arachidonic acid into inflammatory prostaglandins (PGs). COX-2 plays a role in inflammation via the arachidonic acid pathway, producing prostaglandins E2 (PGE2) and I2 (PGI2) and influencing bone remodeling. Therefore, COX-2 activity needs to be inhibited to prevent the formation of osteoclasts and a decrease in bone mass [6,7,8].

Natural compounds from medicinal plants, including flavonoids, exhibit antioxidant and anti-inflammatory activities. In addition, isoflavones (a subclass of flavonoids), as the main group of phytoestrogens, play a strong role as antioxidants and anti-inflammatory agents to prevent osteoporosis [9,10]. The World Health Organization (WHO) has continuously supported medicinal plant research through ethnopharmacological investigations [11,12,13].

Previous investigations have shown that the Leguminosae/Fabaceae family is the most dominant plant examined as having anti-osteoporosis effects [14]. Another study has been conducted on the extraction of *Spatholobus littoralis* Hassk. stems (Leguminosae family) and *Sauropus androgynus* L. (Phyllanthaceae family) leaves utilizing Response Surface Methodology–Central Composite Design (RSM-CCD). The solvents utilized were 70% ethanol and ethyl acetate:*n*-hexane (1:1). The extraction optimization showed that the optimum solid-to-solvent ratio and temperature were 1:15 and 39 to 40 °C, respectively. The ethanol extract from *S. androgynus* L. leaves, prepared with 70% ethanol, achieved the highest yield and TFC, measuring 37.51 ± 0.43% and 25.78 ± 0.36 mg QE/g dry weight (DW), respectively. The 70% ethanol extract of *S. littoralis* Hassk. stems achieved the highest TPC value of 26.64 ± 0.36 mg GAE/g DW. Additionally, profiling analysis was conducted employing GC-MS and LC-MS/M. The analysis reported that phytoestrogens and estrogenic compound groups, such as fatty acids, phenolics, carboxylic acids, and flavonoids, were abundantly contained in the optimum extract obtained. This group of compounds plays a role in bone development [15].

Based on previous research, the optimum extract possesses antioxidant and anti-inflammatory activities closely related to high TFC and TPC values. The optimum formula must be analyzed from the optimum extract combination obtained. Therefore, this study aimed to optimize the formulation of *S. littoralis* Hassk. and *S. androgynus* L. extracts using RSM-SLD as an antioxidant and anti-inflammation agent for osteoporosis treatment. The response used was DPPH (2,2-diphenyl-1-picrylhydrazyl) antioxidant activity, with the factor being the optimal extract concentration. The optimum formula obtained was investigated in vitro and in silico for COX-2 (inflammatory mediator) inhibitory activity. The formulation as an antioxidant and anti-inflammation agent is a novelty in this research.

## 2. Materials and Methods

### 2.1. Plant Collection

*S. littoralis* Hassk. and *S. androgynus* L. were taken from Cipurut, Sukabumi Regency, West Java Province, and Muara Teweh, North Barito Regency, Central Kalimantan Province, respectively. These plants were identified by a botanist from the National Research and Innovation Agency (BRIN) in Cibinong, West Java, Indonesia, using the codes B-1395/II.6.2/IR.01.02/5/2024 and B-2953/II.6.2/IR.01.02/8/2024.

### 2.2. Formulation Optimization Using RSM-SLD

In this study, the extract was diluted with solvents specific to the solvent used, 70% ethanol and ethyl acetate:*n*-hexane. Ethanol and ethyl acetate are classed as Class 3 solvents, which means they are low in toxicity and pose a lower danger to human health. While *n*-hexane is classed as Class 2, its toxicity is less severe, and solvent residues should be kept to a minimum to maintain human health. The use of these solvents did not affect the results of the biological activity tests. The results of the biological activity tests were influenced by the active compound content of the extract obtained [16,17].

Optimization of the formulation of the four extracts, including 70% ethanol extract of *S. littoralis* Hassk. stems, 70% ethanol extract of *S. androgynus* L. leaves, ethyl acetate:*n*-hexane (1:1) extract of *S. littoralis* Hassk. stems, and ethyl acetate:*n*-hexane (1:1) extract of *S. androgynus* L. leaves was carried out using the RSM method combined with the SLD 2-replicate on Design Expert software 13 (Table 1). The concentration of each extract was entered as a combination factor, while the response used was the inhibitory power against DPPH. A one-sample *t*-test was used to confirm the optimization results.

### 2.3. Determination of DPPH Antioxidant Activity

Antioxidant activity was carried out using the DPPH method, referring to research conducted by Rafi et al. [18]. A 125 mM DPPH solution was prepared by dissolving 5 mg of DPPH in 100 mL of pro-analyst ethanol. Meanwhile, the 10,000 ppm concentration sample solution was prepared by dissolving 10 mg of extract in 1 mL of DMSO with a sonicator. The sample solution was diluted to the 1000 ppm concentration range. The test involved mixing 100 μL of the sample with 100 μL of DPPH, while the blank contained 100 μL of ethanol with 100 μL of DPPH. The positive control was ascorbic acid, operated within a concentration range of 0.625–20 ppm. Incubation took place in the darkness for 30 min at room temperature. Absorbance was recorded at 517 nm through a microplate reader (Epoch-BioTek, Winooski, VT, USA). The DPPH antioxidant activity test was repeated three times. Antioxidant activity was calculated using Equation (1):

(1) $$\%\;inhibition\;of\;DPPH=\left[\frac{\left(Corrected\;blank\;absorbance\;\;-Corrected\;sample\;absorbance\right)}{\left(Corrected\;blank\;absorbance\right)}\times 100\%\right]$$

### 2.4. Model Verification

Formulation optimization based on the concentration of each extract factor in producing optimum DPPH antioxidant activity values was verified with %RSD (relative standard deviation) and %RSE (residual standard error) in Equations (2) and (3).

(2) $$\%RSD=\frac{standard\;deviation}{mean}\times 100\%$$

(3) $$\%RSE=\frac{\left(actual\;value-predicted\;value\right)}{predicted\;value}\times 100\%$$

### 2.5. Determination of Extract Toxicity and Optimum Formula Using the BSLT Method

The BSLT method is presented by Meyer et al. [19]. This method started with the hatching of *Artemia salina* L. eggs. A total of 50 mg of *A. salina* L. eggs was weighed and placed in a container filled with clean seawater and left for 48 h under light to hatch perfectly. The hatched larvae were used in the toxicity assay. Subsequently, 10 *A. salina* L. larvae were placed in a 24-well microplate containing seawater, and the extract solution was added to obtain final concentrations of 1000, 500, 100, and 50 ppm with three repetitions. The number of dead larvae was counted to determine the outcome. Cumulative mortality percentage data were processed through probit analysis or lethal concentration 50 (LC_50_) using the IBM SPSS Statistics version 25 program at a 95% confidence interval.

### 2.6. Determination of Inhibition of Extract Optimum and Optimum Formula Against COX-2 Activity (In Vitro)

The COX-2 inhibition analysis is presented in the study by Tuwalaid et al. [20]. In vitro inhibition of COX-2 activity was assessed using the COX-2 (Human) Inhibitor Screening Assay kit (Cayman Chemical, Item no. 701080). The optimal extract and formulation were selected for the COX-2 inhibition test. The procedure followed the kit’s instructions, including reagent preparation, the reaction process, and the assay steps, and ELISA measurements were performed at 412 nm. COX-2 inhibition was calculated using Equation (4).

(4) $$\%inhibition=\frac{(\left[PG\right]corrected\;sample-\left[PG\right]corrected\;of\;100\%\;initial\;activity\;samples}{\left[PG\right]corrected\;of\;100\%\;initial\;activity\;samples}\times 100\%$$

### 2.7. Statistical Analysis

The data were presented as the average of three replicates ± standard deviation and statistically analyzed using a one-way ANOVA. Subsequently, Tukey’s test was applied at a 95% confidence interval, with *p*-values ≤ 0.05 indicating substantial differences between the populations tested.

### 2.8. In Silico Methods

#### 2.8.1. Validation of Molecular Docking: Re-Docking

Re-docking was performed using Ubuntu 22.04.3; Vina 1.2.6, which was conducted based on a script. The procedure included docking the co-crystal ligands present in the receptor. The results were the coordinate center, affinity energy, and RMSD of the flexible ligand. Furthermore, the average coordinate center result was used as a reference for cross-docking. An ANOVA was performed on the RMSD of the flexible ligand, and RMSD values less than 2.0 Å were adopted in interpreting molecular docking validation.

#### 2.8.2. Cross-Docking

Cross-docking was performed using Ubuntu 22.04.3; Vina 1.2.6, based on a script. The process comprised docking co-crystal ligands from all receptors. Cross-docking was carried out to determine the amino acid residues between the ligand and the receptor.

#### 2.8.3. Analysis of LigPlot+

LigPlot+ analysis was performed to obtain data on interactions between ligands and receptors during docking. This included hydrogen bonds and hydrophobic interactions during re-docking and cross-docking.

#### 2.8.4. Ligand Preparation

The ligands used in this study were 80 putative compounds contained in 70% ethanol extract of *S. littoralis* Hassk. stems and 70% ethanol extract of *S. androgynus* L. leaves as the optimum formula. The content of these compounds is suspected to be present in the optimum formula. This is a limitation in this study. Metabolite profiles were previously identified using GC-MS and LC-MS/MS. The control ligand was a co-crystal ligand obtained from the six ligands. All 3D ligand structures were retrieved from PubChem at the National Center for Biotechnology Information (NCBI) website at https://pubchem.ncbi.nlm.nih.gov/ (accessed on 4 March 2025) in *.sdf format. These structures were then converted to *.pdb format employing PyMOL software 3.0.3 and charged with AutodockTools 1.5.6.rc3 before being saved as *.pdbqt files.

#### 2.8.5. Visualization and Interaction of Molecular Docking

Molecular docking between the target receptor and the test ligand was carried out using the oriented process. This was because the protein’s active site was identified through the re-docking step. A total of 20 ligands with strong binding properties were tested for adherence to Lipinski’s rules and ADMET (absorption, distribution, metabolism, excretion, and toxicity) using the admetSAR 2.0, ADMETlab 2.0, SwissADME (https://www.swissadme.ch/), and PROTOX 3.0 applications. Further docking visualization and interactions were performed through LigPlot+ and Discovery Studio Visualizer in 2D and 3D on the best tested ligands.

## 3. Results and Discussion

### 3.1. Formulation Optimization: Effect of Optimum Extract Concentration Factor on DPPH Antioxidant, Model Fitting, and Verification of Extraction Optimization

**Figure 1:**
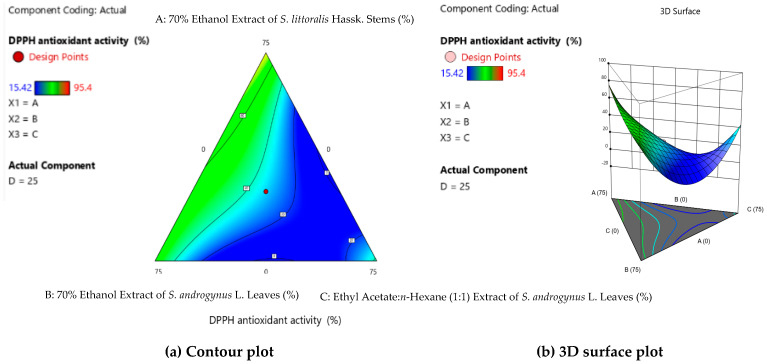
Contour plot (**a**) and 3D surface plot (**b**) for formulation optimization.

Table 2 shows the effect of the optimum extract concentration factor that affects DPPH antioxidant activity. Formula (10) produced a maximum DPPH antioxidant of 95.40 ± 0.78%^a^. Formula (10) consists of 70% ethanol extract of *S. littoralis* Hassk. stems (E1) and 70% ethanol extract of *S. androgynus* L. leaves (E2) (1:1). Figure 1a,b, showing the contour and 3D surface plots for formulation optimization, indicate that the green color in compositions E1 and E2 results in the highest DPPH antioxidant activity. In the formulation or combined extracts, activity has a greater value than the single extract. However, the combined formulations of E1 and E3, E1 and E4, E2 and E3, and E2 and E4 produce lower DPPH antioxidant activity than the single extracts.

Table 3 reports the ANOVA results for formulation optimization. Table 4 presents the model regression data and coded equations for the DPPH antioxidant activity response. The regression model recommended by the ANOVA is the special cubic model. This model shows that the use of three factors or three types of extract will increase the antioxidant activity of DPPH. ANOVA parameters can also be observed from the *p*-value, R^2^, adjusted R^2^, predicted R^2^, and adequate precision. The fourth factor, the type of extract in the experimental design model, had a significant effect, as indicated by a *p*-value < 0.05. Meanwhile, the lack of fit was not significant (*p*-value > 0.05), meaning that the model predictions were not significantly different from the observations, and the constructed experimental model fits the data in the observed replication variations. R^2^ is a measure of the accuracy with which a model explains variance around the mean. Goodness of fit is determined by an R^2^ value greater than 0.95 for all responses, indicating the highest similarity between the predicted and actual responses [21,22,23].

The optimum formula conditions obtained in RSM-SLD were 70% ethanol extract of *S. littoralis* Hassk. stems (E1) and 70% ethanol extract of *S. androgynus* L. leaves (E2) or both at a ratio of 1:1. This optimum formula condition has a DPPH antioxidant activity of 94.78% ± 1.12% with an IC_50_ value of 108.70 µg/mL. Verification of the formulation optimization can be observed in Table 5. The %RSD value is frequently used to verify optimization, implying that each response test is precisely repeatable. The %RSD obtained in this investigation was less than 5%, indicating moderate precision (moderate %RSD) and that the predicted value was not significantly different from the actual value. The %RSE value is further verified by a *p*-value > 0.05 from the one-sample *t*-test, indicating that the actual value obtained is near to and not substantially different from the predicted value given by RSM-SLD. This optimum verification has a desired value close to 1000, which indicates that the optimum conditions of the formula recommended by the combined RSM-SLD are close to the predicted response value (DPPH antioxidant activity) [23,24,25,26].

### 3.2. Using Brine Shrimp Lethality Test (BSLT) to Determine Toxicity of Optimum Extract and Formula

**Figure 2:**
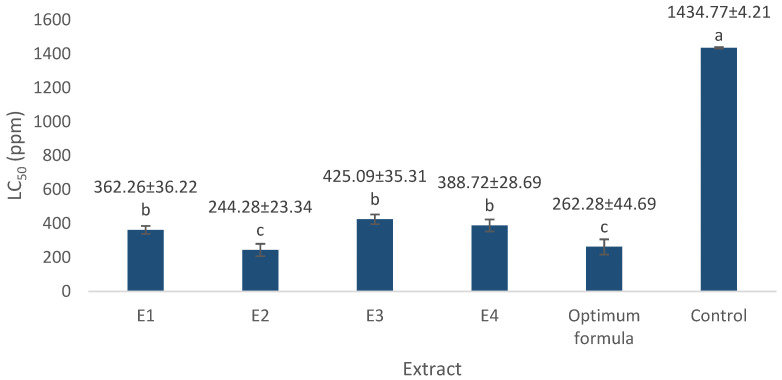
LC_50_ value of optimum extract and formula. E1 = 70% ethanol extract of *S. littoralis* Hassk. stems; E2 = 70% ethanol extract of *S. androgynus* L. leaves; E3 = ethyl acetate:*n*-hexane (1:1) extract of *S. littoralis* Hassk. stems; E4 = ethyl acetate:*n*-hexane (1:1) extract of *S. androgynus* L. leaves; optimum formula = extract combination of E1:E2 (1:1). For each sample, values with different letters in the same column indicate significant differences at *p*-value ≤ 0.05 based on a one-way ANOVA followed by Tukey’s test (*n* = 3). Results are sorted in ascending order: a > b > c.

Figure 2 shows the toxicity analysis results for the extract and optimum formula. The LC_50_ value is used to determine the concentration in the COX-2 inhibition activity test because the value is regarded as safer in drug formulations when the concentration is less than the LC_50_ value [27,28]. The LC_50_ value of the extract and optimum formula is below 1000 mg/L due to toxic *A. salina* L. [19]. A lower LC_50_ value indicates that the extract has a high pharmacological effect, while a high LC_50_ value indicates a low pharmacological effect in the extract. The LC_50_ value for the 70% ethanol extract of *S. androgynus* L. (E2) leaves is lower than that of the other three optimum extracts and is still in the same LC_50_ value group as the optimum formula based on the results of Tukey’s assay. This indicates that E2 contains more secondary metabolites and is therefore more toxic to *A. salina* L. [29,30]. This is in line with the secondary metabolite profiles analyzed using GC-MS and LC-MS/MS in our previous study. We identified 45, 47, 33, and 42 putative compounds in E1, E2, E3, and E4, respectively. The majority of compounds contained in the four extracts were fatty acids (16.5%), phenolics (14.8%), carboxylic acids (13.9%), and flavonoids (12.2%) [15].

Toxic compounds in the extract and optimal formulation may enter *A. salina* L. through the mouth and be absorbed in the digestive tract through membrane cells. This leads to the toxin’s distribution within *A. salina* L., triggering metabolic reactions. Metabolic damage occurs rapidly and can be observed within 24 h, resulting in a 50% mortality rate. The larvae’s mortality rate is closely connected to the concentration and synergistic effects of various compounds in the extract [31,32].

### 3.3. Inhibitory Activity of Extract and Optimum Formula Against COX-2

The COX-2 inhibition assay, based on the Cayman Chemical Item no. 701,080 kit, relies on competition between free prostaglandin (PG) and the PG-AChE conjugate (PG tracer) for binding to a limited amount of antiserum. The PG tracer concentration remains constant, but the free PG varies because the amount of tracer bound by the antiserum is inversely related to its concentration in the well plate. Subsequently, the rabbit antiserum–PG complex attaches to a mouse monoclonal anti-rabbit antibody previously added to the well plate, which is washed to terminate any unbound reagents. Ellman’s reagent, containing acetylcholine as a substrate for AChE, is added. The enzymatic reaction produces a yellow-colored product with an absorbance peak at 412 nm. The spectrophotometric measurement of the color intensity correlates directly with the amount of PG tracer attached to the well plate.

**Figure 3:**
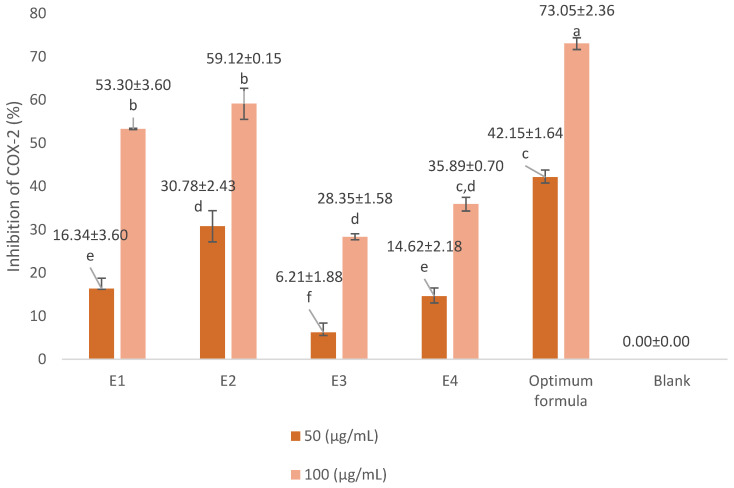
Inhibitory activity of extract and optimum formula against COX-2. E1 = 70% ethanol extract of *S. littoralis* Hassk. stems; E2 = 70% ethanol extract of *S. androgynus* L. leaves; E3 = ethyl acetate:*n*-hexane (1:1) extract of *S. littoralis* Hassk. stems; E4 = ethyl acetate:*n*-hexane (1:1) extract of *S. androgynus* L. leaves; optimum formula = extract combination of E1:E2 (1:1). For each sample, values with different letters in the same column indicate significant differences at *p*-value ≤ 0.05 based on a one-way ANOVA followed by Tukey’s test (*n* = 3). Results are sorted in ascending order: a > b > c > d > e > f.

**Figure 4:**
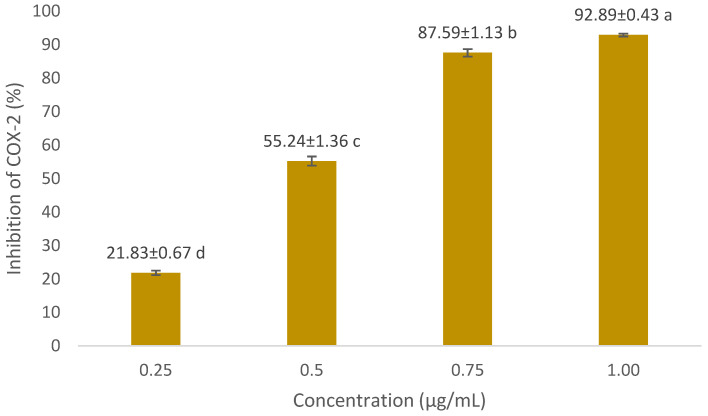
COX-2 inhibitory activity of diclofenac (positive control) against COX-2 at various concentrations. For each sample, values with different letters in the same column indicate significant differences at *p*-value ≤0.05 based on a one-way ANOVA followed by Tukey’s test (*n* = 3). Results are sorted in ascending order: a > b > c > d.

In COX-2 inhibition investigations, the optimum concentration of extract and formula is below the LC_50_ value. We used concentrations of 50 µg/mL and 100 µg/mL. The COX-2 inhibition assay results are presented in Figure 3 and Figure 4. The positive control we used was diclofenac, a synthetic drug commonly used in anti-inflammatory drugs. Diclofenac’s inhibitory power was higher than that of the extract and the optimum formula. At a concentration of 0.75 µg/mL, diclofenac was able to inhibit 87.59 ± 1.13% of COX-2 activity. The optimum formula had the greatest COX-2 inhibitory power (73.05 ± 2.36% at 100 µg/mL) compared to the single extract. Meanwhile, for the single extract, 70% ethanol extract of *S. androgynus* L. leaves (E2) had the highest inhibitory power among the other single extracts, which was 59.12 ± 0.15% at a concentration of 100 µg/mL. This strong COX-2 inhibition is related to the concentration of the extract as an inhibitor will increase the chances of the extract binding to the active site of the COX-2 enzyme, thereby inhibiting COX-2 activity. Flavonoid compounds are often reported as COX-2 inhibitors [20,33,34]. Shamsudin et al. [35] and Alharbi et al. [36] identified quercetin as the strongest COX-2 inhibitor. Quercetin belongs to the flavonol class of flavonoids. The existence of double bonds between C2 and C3 at the C-ring, and the hydroxylation of C3′ and C4′ at the B-ring, were essential to COX-2 inhibition.

Research related to in vitro COX-2 inhibition of single extracts from *S. littoralis* Hassk. and *S. androgynus* L. has not been conducted before. However, Maskur et al. [37] reported that ethanol extract of *S. littoralis* Hassk. could inhibit 43.28% protein denaturation at a concentration of 50 ppm with an IC_50_ of 77.92 µg/mL in an in vitro study. In addition, in an in vivo study, methanol extract of *S. androgynus* L. can reduce inflammation by 91.0% in a carrageenan-induced rat model with an effective dose of 37.80 mg/200 g body weight (BW) [38]. Meanwhile, at the highest dose of 400 mg/kg BW, a 96% ethanol extract of *S. littoralis* Hassk. can suppress 87.65% of inflammation in carrageenan-induced male rats [39]. In vitro and in vivo studies on both plants are in line with in vitro data on COX-2 inhibition from the optimum formula (combination of *S. littoralis* Hassk. and *S. androgynus* L.) presented in this study, showing that *S. littoralis* Hassk. and *S. androgynus* L. act as anti-inflammatory agents.

### 3.4. Molecular Docking Analysis

**Figure 5:**
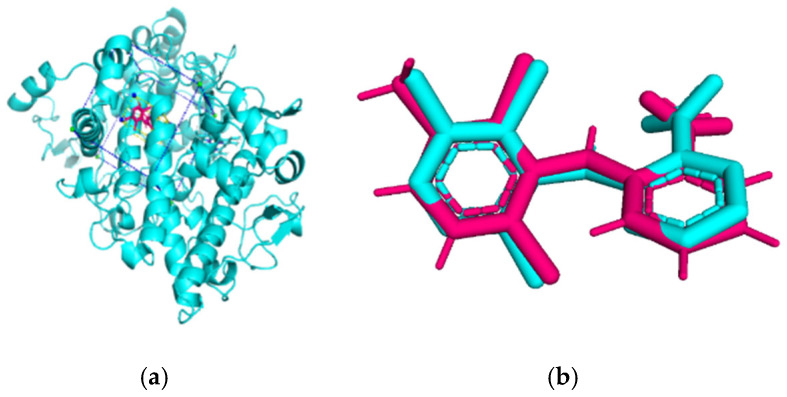
Visualization (**a**) and validation of molecular docking on COX-2 (PDB ID 5IKQ) (re-docking) (**b**). In (**a**), the blue color is a receptor (5IKQ), and the pink color is a ligand (JMS). Then, in (**b**), the blue color is the ligand before re-docking, and the pink color is the ligand after re-docking.

Validation of molecular docking (re-docking) is carried out by re-docking the receptor with its co-crystal ligand with the aim of determining the docking coordinates (center coordinates) in the grid box. These docking coordinates are the binding sites between the co-crystal ligand and its receptor. Molecular docking parameters can be observed in Table 6. The RMSD (Root Mean Square Deviation) value of re-docking, which is less than 2.0 Å, indicates the reliability and robustness of the molecular docking analysis carried out and the small deviation of errors in molecular docking [40,41]. We can observe the re-docking visualization in Figure 5a. Re-docking with three repetitions of receptors that play a role in inflammation (COX-2) in this study obtained an RMSD of <2.0 Å (Figure 5b).

The total of 80 putative compounds obtained from 70% ethanol extract of *S. littoralis* Hassk. stems and 70% ethanol extract of *S. androgynus* L. leaves were docked to the 5IKQ receptor using the docking coordinates during re-docking. This step is called cross-docking. We obtained five compounds as potent inhibitors of COX-2 receptor binding (Table 7). These five compounds come from five groups of compounds: flavonoids, fatty acids, terpenoids, amino acid derivatives, and amine derivatives. The strongest compounds found in each of the five chemical groups were daidzein (−8.514 kcal/mol); (10E,12Z)-9-Hydroperoxy-10,12-octadecadienoic acid (−7.440 kcal/mol); arteannuic acid (−7.114 kcal/mol); L-Proline,4-hydroxy-5-oxo-4-(tetrahydro-2,3,4-trihydroxy-2-furanyl)- (−6.480 kcal/mol); and Androst-2-en-17-amine,4,4-dimethyl-N-(2-phenylethyl)-, (5.alpha.)- (−5.440 kcal/mol). Additionally, they were in line with ADMET and Lipinski’s rule, indicating that they are safe options for oral bioavailability in human consumption [42,43].

Daidzein is sourced from a 70% ethanol extract of *S. littoralis* Hassk. stems. Daidzein belongs to the isoflavone group (flavonoids), which is the main group of phytoestrogens. Therefore, this compound resembles 17ß-estradiol in structure and activates both estrogen receptor alpha (ER-α) and beta (ER-β). The binding affinity of daidzein in binding COX-2 in this in silico study is almost close to the binding affinity strength of the COX-2 co-crystal ligand. The high binding affinity of daidzein has the potential to inhibit inflammation caused by COX-2 activity. Inhibition of COX-2 activity can suppress osteoclastogenesis (bone resorption) [44,45]. (10E,12Z)-9-Hydroperoxy-10,12-octadecadienoic acid is obtained from 70% ethanol extract of *S. androgynus* L. leaves. This compound belongs to the group of polyunsaturated fatty acid compounds. The presence of multiple double bonds and hydroxyl groups in this compound essentially determines its antioxidant, anti-inflammatory, and bone development activities. This compound is responsible for scavenging free radicals and binding to estrogen receptors [46,47]. Arteannuic acid is derived from a 70% ethanol extract of *S. androgynus* L. leaves. This compound belongs to the sesquiterpenoid group (terpenoids). The α,β-unsaturated carboxylic acid group contained in this compound has the potential to act as a free radical scavenger and anti-inflammatory agent [48,49]. L-Proline, 4-hydroxy-5-oxo-4-(tetrahydro-2,3,4-trihydroxy-2-furanyl)- comes from 70% ethanol extract of *S. littoralis* Hassk. stems and is included in amino acid derivatives. Its hydroxyproline content can contribute to bone collagen metabolism and help maintain bone structure [50,51]. Androst-2-en-17-amine,4,4-dimethyl-N-(2-phenylethyl)-, (5.alpha.)- comes from 70% ethanol extract of *S. androgynus* L. leaves and is included in amine derivatives. Antioxidant, anti-inflammatory, and bone development activities from this compound are determined by hydrophobic interaction to bind estrogen receptors, which can contribute to bone development and the presence of a hydroxyl group as an antioxidant and anti-inflammatory agent [52,53]. These five compounds are derived from an optimum formula or extract combination of *S. littoralis* Hassk. stems and 70% ethanol extract of *S. androgynus* L. leaves. The synergistic effect of compounds derived from this optimum formula has been shown to increase COX-2 inhibitory activity and produce high antioxidant activity.

Daidzein and formononetin contained in *S. littoralis* Hassk. were reported by Prasetyorini et al. [54] as bioactive compounds in silico that can inhibit inflammation of the skin (psoriasis). Daidzein was also reported by Rustandi et al. [55] in silico as the strongest antioxidant and anticancer compound. We also found in this study that these compounds have the potential to be anti-inflammatory compounds. Meanwhile, research related to the in silico analysis of compounds from *S. androgynus* L. has previously been reported by Sutjiningsih et al. [56]. They discovered that phytol and other terpenoid chemicals are strong anti-breast cancer agents that attach to the AKT1 protein. Additionally, the findings of these investigations align with our own investigations.

Table 8 and Table 9 show the visualization and types of bonds in cross-docking on six strong COX-2 inhibitors derived from the 70% ethanol extract of *S. littoralis* Hassk. stems and 70% ethanol extract of *S. androgynus* L. leaves. The docking interactions in cross-docking are influenced by hydrogen bonds and hydrophobic interactions. Hydrogen bonds are the attractive forces between hydrogen atoms and those covalently bonded to highly electronegativity atoms, such as nitrogen (N), oxygen (O), and fluorine (F). Hydrogen bonds affect the stability of the receptor–ligand complex. The stability of the ligand relative to the receptor is also determined by hydrophobic interactions. This reduces the interaction between nonpolar amino acid residues and water, leading to degradation of the protein structure and loss of enzyme activity [57].

## 4. Conclusions

In conclusion, RSM-SLD was successfully used to perform formulation optimization. The optimum formula was obtained at the composition of 70% ethanol extract of *S. littoralis* Hassk. stems (E1) and 70% ethanol extract of *S. androgynus* L. leaves (E2) (1:1). This optimum formula has a DPPH antioxidant activity of 94.78% ± 1.12% with an IC_50_ value of 108.70 µg/mL. The optimum formula also has the potential to inhibit inflammatory mediators (COX-2) by 73.05 ± 2.36% at a concentration of 100 µg/mL. The groups of compounds contained in the optimum formula, which have strong affinity for the COX-2 receptor (PDB ID: 5IKQ), are flavonoids, fatty acids, terpenoids, amino acid derivatives, and amine derivatives. In this research, *S. littoralis* Hassk. and *S. androgynus* L. are suggested for use as antioxidant and anti-inflammatory agents for osteoporosis treatment. Future prospects involve analyzing the inhibition of pro-inflammatory cytokine levels in vitro and performing dynamic simulation of in silico analysis. Further research could also be developed using in vivo analysis, such as the carrageenan-induced inflammation method in animal models (mice and rats). The efficacy of formulation development (extract combination of 70% ethanol extract of *S. littoralis* Hassk. stems and 70% ethanol extract of *S. androgynus* L. leaves) could be determined from the development of in vivo methods in the future.

## Figures and Tables

**Table 1:** Combination of formulation optimization factors obtained using RSM-SLD.

Running Order	A: 70% Ethanol Extract of *S. littoralis* Hassk. Stems (% E1)	B: 70% Ethanol Extract of *S. androgynus* L. Leaves (% E2)	C: Ethyl Acetate:*n*-Hexane (1:1) Extract of *S. littoralis* Hassk. Stems (% E3)	D: Ethyl Acetate:*n*-Hexane (1:1) Extract of *S. androgynus* L. Leaves (% E4)
1	0	100	0	0
2	0	50	0	50
3	0	50	50	0
4	12.5	12.5	62.5	12.5
5	25	25	25	25
6	100	0	0	0
7	0	0	50	50
8	50	0	0	50
9	0	0	0	100
10	50	50	0	0
11	50	0	50	0
12	100	0	0	0
13	12.5	62.5	12.5	12.5
14	0	0	100	0
15	62.5	12.5	12.5	12.5
16	12.5	12.5	12.5	62.5
17	0	100	0	0

**Table 2:** RSM-SLD and DPPH antioxidant activity results.

Running Order	A: 70% Ethanol Extract of *S. littoralis* Hassk. Stems (% E1)	B: 70% Ethanol Extract of *S. androgynus* L. Leaves (% E2)	C: Ethyl Acetate:*n*-Hexane (1:1) Extract of *S. littoralis* Hassk. Stems (% E3)	D: Ethyl Acetate:*n*-Hexane (1:1) Extract of *S. androgynus* L. Leaves (% E4)	DPPH Antioxidant Activity ± SD (%)
1	0	100	0	0	78.11 ± 1.31 ^b^
2	0	50	0	50	63.18 ± 1.68 ^d^
3	0	50	50	0	65.17 ± 1.91 ^c,d^
4	12.5	12.5	62.5	12.5	15.42 ± 3.39 ^i^
5	25	25	25	25	31.84 ± 2.40 ^g^
6	100	0	0	0	61.69 ± 1.68 ^d^
7	0	0	50	50	45.40 ± 0.94 ^f^
8	50	0	0	50	55.22 ± 1.63 ^e^
9	0	0	0	100	31.59 ± 2.99 ^g^
10	50	50	0	0	95.40 ± 0.78 ^a^
11	50	0	50	0	50.37 ± 0.99 ^e,f^
12	100	0	0	0	61.94 ± 1.49 ^d^
13	12.5	62.5	12.5	12.5	70.40 ± 1.14 ^c^
14	0	0	100	0	25.12 ± 1.20 ^h^
15	62.5	12.5	12.5	12.5	62.31 ± 1.12 ^d^
16	12.5	12.5	12.5	62.5	35.32 ± 1.14 ^g^
17	0	100	0	0	80.35 ± 2.06 ^b^

For each sample, values with different letters in the same column indicate significant differences at *p*-value ≤ 0.05 based on one-way ANOVA followed by Tukey’s test (*n* = 3). Results are sorted in ascending order: a > b > c > d > e > f > g > h > i.

**Table 6:** Molecular docking parameters.

No.	Receptor	PDB ID	Ligand Code	Grid Box	Spacing	Exhaustiveness	Number of Processors	Center Coordinate of Re-Docking
1.	COX-2	5IKQ	JMS	20 × 20 × 20 Å	0.375	32	16	[22.228, 51.433, 17.644]

**Table 7:** The 5 strong compounds that bind to COX-2 (PDB ID: 5IKQ) as anti-inflammatory agents.

Receptor	PDB ID	Co-Crystal Ligand	ΔG (kcal/mol)	No.	Group of Compounds	Compounds	PubChem ID	ΔG (kcal/mol)	Source
COX-2	5IKQ	JMS	−9.109	1	Flavonoids (isoflavone)	Daidzein	CID_5281708	−8.514	70% ethanol extract of *S. littoralis* Hassk. stems
				2	Fatty acids (polyunsaturated fatty acids)	(10E,12Z)-9-Hydroperoxy-10,12-octadecadienoic acid	CID_6439847	−7.604	70% ethanol extract of *S. androgynus* L. leaves
				3	Terpenoids (sesquiterpenoid)	Arteannuic acid	CID_10922465	−7.114	70% ethanol extract of *S. androgynus* L. leaves
				4	Amino acid derivatives	L-Proline, 4-hydroxy-5-oxo-4-(tetrahydro-2,3,4-trihydroxy-2-furanyl)-	CID_71437684	−6.480	70% ethanol extract of *S. littoralis* Hassk. stems
				5	Amine derivatives	Androst-2-en-17-amine, 4,4-dimethyl-N-(2-phenylethyl)-, (5.alpha.)-	CID_22296128	−5.440	70% ethanol extract of *S. androgynus* L. leaves

**Table 8:** Visualization of cross-docking.

No.	Receptor–Ligand Complex	Receptor–Ligand Complex Code	PyMoL Visualization	Discovery Studio Visualizer Visualization	LigPlot 3D Visualization	Discovery Studio Visualizer 2D Visualization
1.	COX-2−Daidzein	5IKQ−5281708	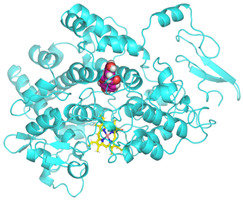	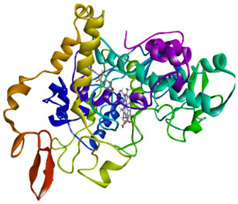	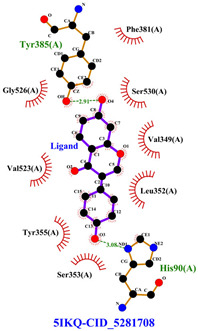	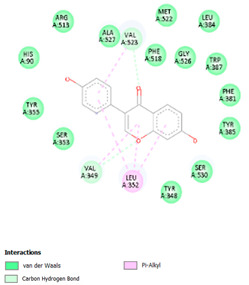
2.	COX-2−(10E,12Z)-9-Hydroperoxy-10,12-octadecadienoic acid	5IKQ−6439847	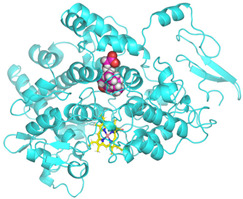	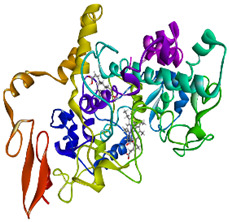	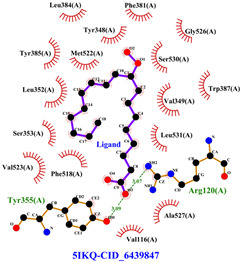	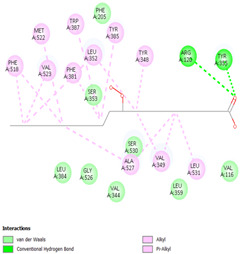
3.	COX-2−L-Proline, 4-hydroxy-5-oxo-4-(tetrahydro-2,3,4-trihydroxy-2-furanyl)-	5IKQ−71437684	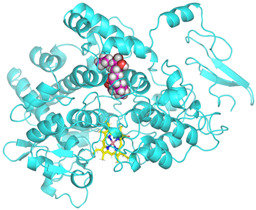	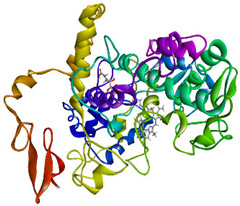	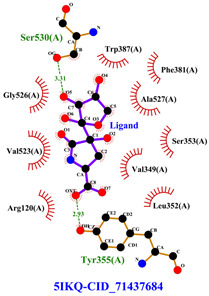	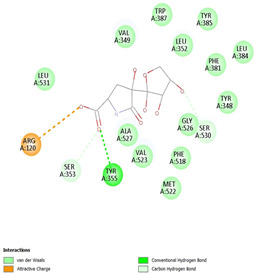
4.	COX-2−Arteannuic acid	5IKQ−10922465	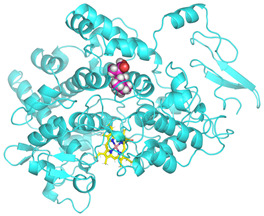	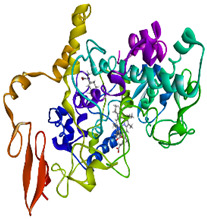	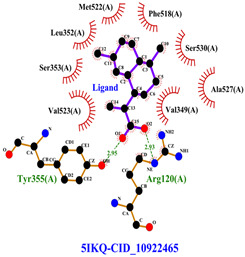	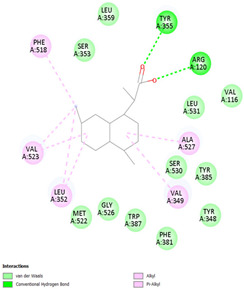
5.	COX-2−Androst-2-en-17-amine, 4,4-dimethyl-N-(2-phenylethyl)-, (5.alpha.)-	5IKQ−22296128	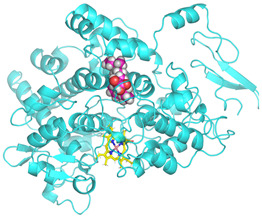	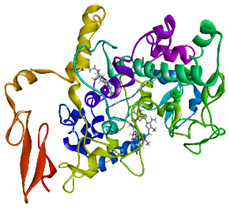	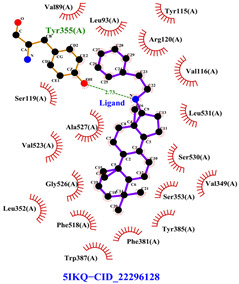	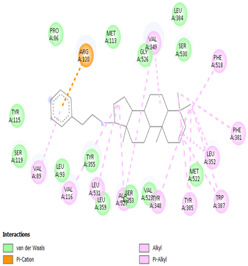

**Table 9:** Types of bonds in cross-docking.

No.	Receptor–Ligand Complex	Receptor–Ligand Complex Code	Hydrogen Bond	Bond Length (Å)	Number of Hydrogen Bonds	Hydrophobic Interactions	Number of Hydrophobic Interactions
1.	COX-2−Daidzein	5IKQ-5281708	Tyr385 His90	2.91 3.08	2	Phe381, Ser530, Val349, Leu352, Ser353, Tyr355, Val523, Gly526	8
2.	COX-2−(10E,12Z)-9-Hydroperoxy-10,12-octadecadienoic acid	5IKQ-6439847	Tyr355 Arg120	3.09 3.07	2	Phe381, Tyr348, Gly526, Ser530, Trp387, Val349, Leu531, Ala527, Val116, Phe518, Val523, Ser353, Leu352, Met522, Tyr385, Leu384	16
3.	COX-2−L-Proline, 4-hydroxy-5-oxo-4-(tetrahydro-2,3,4-trihydroxy-2-furanyl)-	5IKQ−71437684	Ser530 Tyr355	3.31 2.93	2	Trp387, Phe381, Ala527, Val349, Ser353, Leu352, Arg120, Val523, Gly526	9
4.	COX-2−Arteannuic acid	5IKQ−10922465	Arg120 Tyr355	2.93 2.95	2	Met522, Phe518, Ser530, Ala527, Val349, Val523, Ser353, Leu352	8
5.	COX-2−Androst-2-en-17-amine, 4,4-dimethyl-N-(2-phenylethyl)-, (5.alpha.)-	5IKQ−22296128	Tyr355	2.73	1	Val89, Leu93, Tyr115, Arg120, Val116, Leu531, Ser530, Ser353, Val349, Tyr385, Phe381, Trp387, Phe518, Leu352, Gly526, Val523, Ala527, Ser119	18

**Table 3:** ANOVA for the factor of DPPH antioxidant activity.

Source	Sum of Squares	df	Mean Square	F-Value	*p*-Value	
**Model**	7402.46	13	569.42	618.6	<0.0001	significant
Linear Mixture	5443.33	3	1814.44	1971.16	<0.0001	
AB	497.99	1	497.99	541	0.0002	
AC	44.29	1	44.29	48.11	0.0061	
AD	123.55	1	123.55	134.23	0.0014	
BC	53.19	1	53.19	57.78	0.0047	
BD	34.98	1	34.98	38	0.0086	
CD	194.39	1	194.39	211.18	0.0007	
ABC	191	1	191	207.5	0.0007	
ABD	21.56	1	21.56	23.42	0.0168	
ACD	239.01	1	239.01	259.65	0.0005	
BCD	179.15	1	179.15	194.63	0.0008	
**Residual**	2.76	3	0.9205			
Lack of Fit	0.2214	1	0.2214	0.1744	0.7168	not significant
Pure Error	2.54	2	1.27			
**Cor Total**	7405.22	16				
R^2^			0.9996			
Adjusted R^2^			0.998			
Predicted R^2^			0.9411			
Adeq Precision			92.0937			

A: 70% Ethanol Extract of *S. littoralis* Hassk. Stems; B: 70% Ethanol Extract of *S. androgynus* L. Leaves; C: Ethyl Acetate:*n*-Hexane (1:1) Extract of *S. littoralis* Hassk. Stems; D: Ethyl Acetate:*n*-Hexane (1:1) Extract of *S. androgynus* L. Leaves.

**Table 4:** Coded equations in RSM-SLD for the optimum formula.

Extract	Response	Model	Equation
Formulation optimization	DPPH antioxidant activity	Special Cubic	Y = 61.83A + 79.24B + 25.14C + 31.61D + 99.63AB + 27.71AC + 34.17AD + 52.08BC + 31.18BD + 68.26CD − 565.47ABC + 1683.17ABD − 1613.15ACD − 1863.BCD

A: 70% Ethanol Extract of *S. littoralis* Hassk. Stems; B: 70% Ethanol Extract of *S. androgynus* L. Leaves; C: Ethyl Acetate:*n*-Hexane (1:1) Extract of *S. littoralis* Hassk. Stems; D: Ethyl Acetate:*n*-Hexane (1:1) Extract of *S. androgynus* L. Leaves.

**Table 5:** %RSD, %RSE, and one-sample *t*-test on formulation optimization verification.

Factor				Predicted Value	Actual Value			
70% Ethanol Extract of *S. littoralis* Hassk. Stems (E1)	70% Ethanol Extract of *S. androgynus* L. Leaves (E2)	Ethyl Acetate:*n*-Hexane (1:1) Extract of *S. littoralis* Hassk. Stems (E3)	Ethyl Acetate:*n*-Hexane (1:1) Extract of *S. androgynus* L. Leaves (E4)	DPPH Antioxidant Activity (%)	DPPH Antioxidant Activity (%)	%RSD	%RSE	*p*-Value
50	50	0	0	95.44	94.78	1.18	0.70	1.000

## Data Availability

The original contributions presented in this study are included in the article. Further inquiries can be directed to the corresponding author.

## References

[1] Khan J., Gharai P.K., Garg S., Gupta S., Arshi M.U., Mallesh R., Ghosh S. (2025). Discovery of Powerful Multifaceted Antioxidant for Combating Oxidative Stress Associated with Neurodegenerative Disorders. Acta Pharm. Sin. B.

[2] Chaudhary P., Janmeda P., Docea A.O., Yeskaliyeva B., Abdull Razis A.F., Modu B., Calina D., Sharifi-Rad J. (2023). Oxidative Stress, Free Radicals and Antioxidants: Potential Crosstalk in the Pathophysiology of Human Diseases. Front. Chem..

[3] Zhang C., Li H., Li J., Hu J., Yang K., Tao L. (2023). Oxidative Stress: A Common Pathological State in a High-Risk Population for Osteoporosis. Biomed. Pharmacother..

[4] Martemucci G., Costagliola C., Mariano M., D’andrea L., Napolitano P., D’Alessandro A.G. (2022). Free Radical Properties, Source and Targets, Antioxidant Consumption and Health. Oxygen.

[5] Jomova K., Raptova R., Alomar S.Y., Alwasel S.H., Nepovimova E., Kuca K., Valko M. (2023). Reactive Oxygen Species, Toxicity, Oxidative Stress, and Antioxidants: Chronic Diseases and Aging.

[6] Wang C., Ge F., Ge F., Xu Z., Jiang J. (2025). Harnessing Stem Cell Therapeutics in LPS-Induced Animal Models: Mechanisms, Efficacies, and Future Directions. Stem Cell Res. Ther..

[7] Torres H.M., Arnold K.M., Oviedo M., Westendorf J.J., Weaver S.R. (2023). Inflammatory Processes Affecting Bone Health and Repair. Curr. Osteoporos. Rep..

[8] Zhang L., Zheng Y.L., Wang R., Wang X.Q., Zhang H. (2022). Exercise for Osteoporosis: A Literature Review of Pathology and Mechanism. Front. Immunol..

[9] Sharma A.R., Lee Y., Bat-ulzii A., Chatterjee S., Bhattacharya M., Chakraborty C., Lee S. (2023). Bioactivity, Molecular Mechanism, and Targeted Delivery of Flavonoids for Bone Loss. Nutrients.

[10] Barańska A., Kanadys W., Bogdan M., Stępień E., Barczyński B., Kłak A., Augustynowicz A., Szajnik M., Religioni U. (2022). The Role of Soy Isoflavones in the Prevention of Bone Loss in Postmenopausal Women: A Systematic Review with Meta-Analysis of Randomized Controlled Trials. J. Clin. Med..

[11] Zahra M., Abrahamse H., George B.P. (2024). Flavonoids: Antioxidant Powerhouses and Their Role in Nanomedicine. Antioxidants.

[12] Karimi S.M., Bayat M., Rahimi R. (2024). Plant-Derived Natural Medicines for the Management of Osteoporosis: A Comprehensive Review of Clinical Trials. J. Tradit. Complement. Med..

[13] Pradubyat N., Wunnakup T., Praparatana R., Wongwiwatthananukit S., Jongrungruangchok S., Songsak T., Madaka F., Sudsai T. (2024). Evaluation of Antioxidant and Anti-Inflammatory Properties, Bioactive Compound Profiling, and Molecular Mechanisms of a Multicomponent Thai Herbal Formulation. Phytomed. Plus.

[14] Sianipar R.N.R., Iswantini D., Charlena C., Wahyudi S.T., Prasetyo J. (2024). Phytoestrogens Therapy for Osteoporosis Treatment Using Indonesian Medicinal Plants: A Brief Review. Sci. Technol. Indones..

[15] Sianipar R.N.R., Iswantini D., Charlena C., Tri S. (2025). Simultaneous Optimization of Extraction Yield, Flavonoid, and Phenolic Compounds from *Spatholobus littoralis* Hassk and *Sauropus androgynus* L. Using Response Surface Methodology. Iran. J. Chem. Chem. Eng. (IJCCE).

[16] Cendrowski A., Studnicki M. (2024). Applied Sciences Impact of Different Solvents and Temperatures on the Extraction of Bioactive Compounds from Rose Fruits (*Rosa rugosa*) Pomace. Appl. Sci..

[17] Plaskova A., Mlcek J. (2023). New Insights of the Application of Water or Ethanol-Water Plant Extract Rich in Active Compounds in Food. Front. Nutr..

[18] Rafi M., Hasanah T., Karomah A.H., Mulyati A.H., Trivadila, Rahminiwati M., Achmadi S.S., Iswantini D. (2022). FTIR- and UHPLC-Q-Orbitrap HRMS-Based Metabolomics of *Sonchus arvensis* Extracts and Evaluation of Their Free Radical Scavenging Activity. Sains Malays..

[19] Meyer B.N., Ferrigni N.R., Putnam J.E., Jacobsen L.B., Nichols D.E., McLaughlin J.L. (1982). Brine Shrimp: A Convenient General Bioassay for Active Plant Constituents. Planta Med..

[20] Tuwalaid B., Iswantini D., Tri Wahyudi S. (2022). Potential *Adenostemma lavenia* and *Muntingia calabura* Extracts to Inhibit Cyclooxygenase-2 Activity as a Therapeutic Strategy for Anti-Inflammation: Experimental and Theoretical Studies. Indones. J. Chem..

[21] Kamboj A., Chopra R., Singh R., Saxena V., GV P.K. (2022). Effect of Pulsed Electric Field Parameters on the Alkaline Extraction of Valuable Compounds from Perilla Seed Meal and Optimization by Central Composite Design Approach. Appl. Food Res..

[22] Cui L., Ma Z., Wang D., Niu Y. (2022). Ultrasound-Assisted Extraction, Optimization, Isolation, and Antioxidant Activity Analysis of Flavonoids from *Astragalus membranaceus* Stems and Leaves. Ultrason. Sonochem..

[23] Noorulla K.M., Doyo Dalecha D., Jemal Haji M., S R., Arumugam M., Zafar A., Gadisa Gobena W., Mekit S., Haji Negawo H., Hussein M. (2024). Syrupy Herbal Formulation of Green Bean Pod Extract of *Phaseolus vulgaris* L.: Formulation Optimization by Central Composite Design, and Evaluation for Anti-Urolithiatic Activity. Heliyon.

[24] Nurcholis W., Safithri M., Marliani N., Iqbal M. (2023). Response Surface Modeling to Optimize Sonication Extraction with the Maceration Method for the Phenolic Content and Antioxidant Activity of *Justicia gendarussa* Burm f. J. Appl. Pharm. Sci..

[25] Juliana D., Aisyah S.I., Priosoeryanto B.P., Nurcholis W. (2022). Optimization of Cardamom (*Amomum compactum*) Fruit Extraction Using the Box–Behnken Design Focused on Polyphenol Extraction with Antioxidant Activity. J. Appl. Pharm. Sci..

[26] Boateng I.D., Mustapha A., Kuehnel L., Daubert C.R., Kumar R., Agliata J., Flint-Garcia S., Wan C., Somavat P. (2023). From Purple Corn Waste (Pericarp) to Polyphenol-Rich Extract with Higher Bioactive Contents and Superior Product Qualities Using Two-Step Optimization Techniques. Ind. Crops Prod..

[27] Rahminiwati M., Sianipar R.N.R., Sutriah K., Iswantini D., Trivadila T., Achmadi S.S., Sulistyawan I.H. (2023). Optimization of Xanthine Oxidase Activity, Phytochemical Screening, Toxicity Assay, and Antigout Activity of *Spatholobus littoralis* Hassk. Extract. Pharmacogn. J..

[28] Lima L.R., Andrade F.K., Alves D.R., de Morais S.M., Vieira R.S. (2021). Anti-Acetylcholinesterase and Toxicity against Artemia Salina of Chitosan Microparticles Loaded with Essential Oils of Cymbopogon flexuosus, Pelargonium x ssp and Copaifera officinalis. Int. J. Biol. Macromol..

[29] López-Rosas C.A., González-Periañez S., Pawar T.J., Zurutuza-Lorméndez J.I., Ramos-Morales F.R., Olivares-Romero J.L., Saavedra Vélez M.V., Hernández-Rosas F. (2025). Anticonvulsant Potential and Toxicological Profile of *Verbesina persicifolia* Leaf Extracts: Evaluation in Zebrafish Seizure and *Artemia salina* Toxicity Models. Plants.

[30] Sagrin M.S., Lasano N.F., Shukri R., Ramli N.S. (2019). Antioxidant Properties and Toxicity Assessment of the Crescentia Cujete Extracts in Brine Shrimp *(Artemia salina*). Sains Malays..

[31] Fadhli H., Rahmayanı W., Allaya R., Jumadila I.M., Fernando A., Utami R. (2024). Cytotoxic Activity of Isolate Compounds from Derendan (*Lansium parasiticum* (Osbeck) K.C. Sahni & Bennet) Fruit Peel. J. Ilmu Kefarmasian Indones..

[32] Carolina de Almeida M., Machado M.R., Costa G.G., de Oliveira G.A.R., Nunes H.F., Maciel Costa Veloso D.F., Ishizawa T.A., Pereira J., Ferreira de Oliveira T. (2023). Influence of Different Concentrations of Plasticizer Diethyl Phthalate (DEP) on Toxicity of *Lactuca sativa* Seeds, *Artemia salina* and Zebrafish. Heliyon.

[33] Jomova K., Alomar S.Y., Valko R., Liska J., Nepovimova E., Kuca K., Valko M. (2025). Flavonoids and Their Role in Oxidative Stress, Inflammation, and Human Diseases. Chem. Biol. Interact..

[34] Ju Z., Li M., Xu J., Howell D.C., Li Z., Chen F.E. (2022). Recent Development on COX-2 Inhibitors as Promising Anti-Inflammatory Agents: The Past 10 Years. Acta Pharm. Sin. B.

[35] Shamsudin N.F., Ahmed Q.U., Mahmood S., Adnan S., Shah A., Sarian M.N., Muzaffar M., Khan A., Khatib A., Sukarni A. (2022). Flavonoids as Antidiabetic and Anti-Inflammatory Agents: A Review on Structural Activity Relationship-Based Studies and Meta-Analysis. Int. J. Mol. Sci..

[36] Alharbi H.O.A., Alshebremi M., Babiker A.Y., Rahmani A.H. (2025). The Role of Quercetin, a Flavonoid in the Management of Pathogenesis Through Regulation of Oxidative Stress, Inflammation, and Biological Activities. Biomolecules.

[37] Maskur S.K., Tahir M., Razak R. (2025). In Vitro Anti-Inflammatory Activity of Bajakah Tampala Root (*Spatholobus littoralis* Hassk.). Pharm. Rep..

[38] Andari D., Khan F.I., Jakfar S.I. (2022). Methanol Extract of Katuk (*Sauropus androgynus*) Leaves as an Anti-Inflammatory Agent: Animal Study in Carrageenan-Induced Rat Models of Inflammation. Mol. Cell. Biomed. Sci..

[39] Nastati K., Nugraha D.F. (2022). Anti-Inflammatory Activity of Bajakah Wood Extract (*Spatholobus littoralis* Hask). J. Surya Med..

[40] Kashyap M., Gupta S., Bansal Y., Bansal G. (2025). 2D—QSAR Driven Design, Molecular Docking, Molecular Dynamics Simulation and MM/GBSA Studies on Quinazoline Derivatives for Development of VEGFR—2 Inhibitors. Discov. Chem..

[41] Che X., Liu Q., Zhang L. (2023). An Accurate and Universal Protein-Small Molecule Batch Docking Solution Using Autodock Vina. Results Eng..

[42] Wu K., Kwon S.H., Zhou X., Fuller C., Wang X., Vadgama J., Wu Y. (2024). Overcoming Challenges in Small-Molecule Drug Bioavailability: A Review of Key Factors and Approaches. Int. J. Mol. Sci..

[43] Ibeyaima A., Loying R., Manna P. (2025). Prediction of the Toxicity, Bioavailability, Pharmacokinetics, and Lipinski Rule of 5 in the Antidiabetic Compounds: A Computer Based Investigation. Silico Res. Biomed..

[44] Jayusman P.A., Nasruddin N.S., Baharin B., Ibrahim N.I., Ahmad Hairi H., Shuid A.N. (2023). Overview on Postmenopausal Osteoporosis and Periodontitis: The Therapeutic Potential of Phytoestrogens against Alveolar Bone Loss. Front. Pharmacol..

[45] Bellavia D., Dimarco E., Costa V., Carina V., De Luca A., Raimondi L., Fini M., Gentile C., Caradonna F., Giavaresi G. (2021). Flavonoids in Bone Erosive Diseases: Perspectives in Osteoporosis Treatment. Trends Endocrinol. Metab..

[46] Feehan O., Magee P.J., Pourshahidi L.K., Armstrong D.J., Slevin M.M., Allsopp P.J., Conway M.C., Strain J.J., McSorley E.M. (2023). Associations of Long Chain Polyunsaturated Fatty Acids with Bone Mineral Density and Bone Turnover in Postmenopausal Women. Eur. J. Nutr..

[47] Murthy H.N., Yadav G.G., Joseph K.S., HS S.K., Magi S.M., Dewir Y.H., Mendler-Drienyovszki N. (2024). Nutritional Value, Fatty Acid and Phytochemical Composition, and Antioxidant Properties of Mysore Fig (*Ficus drupacea* Thunb.) Fruits. Foods.

[48] Mantiniotou M., Athanasiadis V., Kalompatsios D., Bozinou E., Lalas S.I. (2025). Therapeutic Capabilities of Triterpenes and Triterpenoids in Immune and Inflammatory Processes: A Review. Compounds.

[49] Gao T., Yu C., Shi X., Hu Y., Chang Y., Zhang J., Wang Y., Zhai Z., Jia X., Mao Y. (2024). Artemisinic Acid Attenuates Osteoclast Formation and Titanium Particle-Induced Osteolysis via Inhibition of RANKL-Induced ROS Accumulation and MAPK and NF-ΚB Signaling Pathways. Front. Pharmacol..

[50] Wang J., Hou H., Li Y., Tang W., Gao D., Liu Z., Gao X.Q., Zhao F., Sun F., Tan H. (2024). Isolation, Purification, and Antiosteoporosis Activity of Donkey Bone Collagen from Discarded Bone and Its Antioxidant Peptides. Heliyon.

[51] Hao Y., Xing L., Wang Z., Cai J., Toldrá F., Zhang W. (2023). Study on the Anti-Inflammatory Activity of the Porcine Bone Collagen Peptides Prepared by Ultrasound-Assisted Enzymatic Hydrolysis. Ultrason. Sonochem..

[52] Chen Y.J., Jia L.H., Han T.H., Zhao Z.H., Yang J., Xiao J.P., Yang H.J., Yang K. (2024). Osteoporosis Treatment: Current Drugs and Future Developments. Front. Pharmacol..

[53] Huang S.K.H., Bueno P.R.P., Garcia P.J.B., Lee M.J., De Castro-Cruz K.A., Leron R.B., Tsai P.W. (2023). Antioxidant, Anti-Inflammatory and Antiproliferative Effects of *Osmanthus fragrans* (Thunb.) Lour. Flower Extracts. Plants.

[54] Prasetyorini B.E., Kusumawardani A., Fitriani F., Rachman P.O., Amelinda N., Ramadhani A. (2022). Analisis In Silico Senyawa Aktif Batang Kayu Bajakah (*Spatholobus Littoralis* Hassk) Sebagai Terapi Psoriasis. Herb-Med. J..

[55] Rustandi T., Yumassik A.M., Nugraha E.J.E.P., Nugroho M.R., Yasir M. (2025). Kamalia Antioxidant and Anticancer Activities of Spatholobus Littoralis Stem Extract: An In Vitro and In Silico Computational Investigation. J. Food Pharm. Sci..

[56] Sutjiningsih N.N.O., Ulisya A.A., Utami A., Natalia C., Mumtaz F.C., Yulanda N.R.E., Sari V.R., Auli W.N., Saputro A.H. (2025). Molecular Docking of Several Compounds in Katuk (*Sauropus androgynus* (L.)) Leaves as Anti-Breast Cancer in AKT1 Protein. Pharm. Rep..

[57] Vaidyanathan R., Murugan Sreedevi S., Ravichandran K., Vinod S.M., Hari Krishnan Y., Babu L.K., Parthiban P.S., Basker L., Perumal T., Rajaraman V. (2023). Molecular Docking Approach on the Binding Stability of Derivatives of Phenolic Acids (DPAs) with Human Serum Albumin (HSA): Hydrogen-Bonding versus Hydrophobic Interactions or Combined Influences?. JCIS Open.

